# Tunable Properties of Non‐Volatile Magnetic Mixtures on Different Surfaces

**DOI:** 10.1002/cphc.202400458

**Published:** 2024-11-03

**Authors:** Sergio J. Abellán‐Martín, Cristina Zapater, Nerea González‐Gallardo, Miguel Ángel Aguirre, Lorena Vidal, Diego J. Ramón, Antonio Canals

**Affiliations:** ^1^ Department of Analytical Chemistry Nutrition and Food Science and University Institute of Materials Faculty of Science University of Alicante P.O. Box 99 03080 Alicante Spain; ^2^ Department of Organic Chemistry and University Institute of Organic Synthesis (ISO) Faculty of Science University of Alicante P.O. Box 99 03080 Alicante Spain

**Keywords:** Glass materials, Polypropylene materials, Magnetic Deep Eutectic Solvents (MDES), Magnetic Low Transition Temperature Mixtures (MLTTM), Surface interactions

## Abstract

In this work, the surface nature‐dependent behaviors of magnetic deep eutectic solvents (MDESs) and magnetic low‐transition‐temperature non‐volatile mixtures (MLTTMs) are reported for the first time. It has been observed that the surface of the material where the MDES or the MLTTM is placed could considerably affect the dispersion and the magnetic and structural properties of these magnetic mixtures. Different techniques and analyses have been carried out to highlight the differences observed in the properties depending on the material on which these magnetic mixtures are placed. To that end, differential scanning calorimetry (DSC), surface tension, contact angle and scanning electron microscopy with energy dispersive X‐ray spectroscopy (SEM‐EDX) measurements have been performed. As a result, it has been shown that the MDESs or MLTTMs are retained and adhered to glass surfaces, resulting in a loss of magnetism of the mixture in addition to a loss in the performance of synthesis carried out on the closeness of glass materials as the interaction between the glass and the mixture modify the composition and therefore the properties. As a preliminary result, when using these magnetic mixtures as extractant solvents in dispersive liquid‐liquid microextraction, the MDES or MLTTM is retained on the walls of the glass tubes reducing the extraction efficiency, repeatability and the extraction recovery using an external magnetic field. For all these reasons, polypropylene materials should be recommended when handling magnetic deep eutectic solvents and non‐volatile MLTTMs.

## Graphical abstract

Surface nature‐dependent adhesion behavior has been observed in magnetic non‐volatile mixtures. As a result, a loss in magnetic properties and in the ability to be used as an extractant solvent has been observed on glass surface. For this reason, it is of great importance to take into account the material used when using these new solvents. Furthermore, we need to reconsider the materials used in the past when synthesizing and using these mixtures.

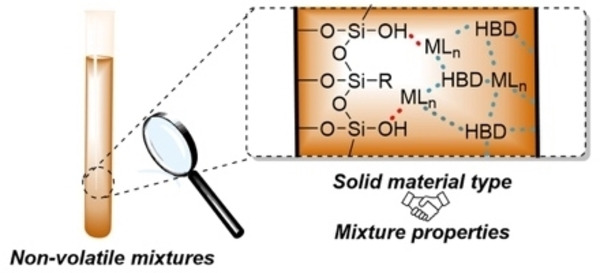

## Introduction

Deep eutectic solvents (DESs) were introduced as a promising alternative to conventional solvents and to ionic liquids (ILs) by Abbot et al. for the first time in 2004.^[1]^ DESs were described as mixtures of two (or more) pure components, Lewis and Bronsted acids and bases generally, with a eutectic point temperature much lower than its individual components.[^1^, ^2^, ^3^] This intense depression in the melting point has been mainly attributed to strong hydrogen bonds along with (if applicable) electrostatic interactions which hinder the possibility of forming a well‐defined crystalline network leading to mixtures that are liquids at room temperature.^[4]^ Traditionally, these systems have been prepared by mixing quaternary ammonium salts and hydrogen bonds donors (HBDs) such as alcohols, amines or carboxylic acids.[^1^, ^2^, ^3^] Besides DES, low‐transition‐temperature mixture (LTTM) is another term extensively used in this field. The main difference between LTTM and DES is that the latter shows a well‐defined melting point, whereas the former presents a glass transition point.[^4^, ^5^, ^6^] Although, any non‐volatile LTTM could behave as these mixtures (i. e., DES) for practical purposes.^[7]^

In recent years, a special DES category containing magnetic components has been gaining attention. In consequence, the preparation of magnetic deep eutectic solvents (MDESs) has significantly increased.[^8^, ^9^, ^10^] Normally, MDESs are composed of HBD, hydrogen bond acceptor (HBA) compounds and an additional magnetic component which is a metal halide (i. e., FeCl_3_, MnCl_2_, NiCl_2_, CoCl_2_ and GdCl_3_) showing an advantageous and potential use in separation processes.[^8^, ^9^] In some cases, MDESs can also be composed of only two components, in which one of them is the magnetic salt (i. e., HBA) and the other component acts as the HBD.^[10]^ The strong susceptibility that MDESs show to external magnetic fields is significantly advantageous in analytical extraction applications, facilitating the phase separation and reducing the number of stages in the sample preparation.[^8^, ^9^, ^11^] However, recently, we have visually observed that the behavior and performance of these magnetic mixtures are heavily influenced by the surface nature of the material in which they are prepared, stored and used (Videos S1–S4). This is significantly important since many applications of these systems rely on their unique yet complex properties and structures. In 2018 it was postulated that due to the ionic nature of these mixtures, their structures could be perturbed in the presence of charged surfaces. This was corroborated by Hammons et al. when they measured the perturbations from curved surfaces of mesoporous silica particles in choline chloride:ethylene glycol (1 : 2) mixtures using synchrotron‐based ultra‐small angle X‐ray scattering (USAXS) experiments to study the solvent distribution near the surface of charged mesoporous silica particles.^[12]^ Nevertheless, to the best of our knowledge, any report has been found in the literature regarding this surface nature‐dependent effect.

In this work, the preliminary results of studies on the preparation and characterization of (novel) different binary and ternary MDESs and magnetic LTTMs (MLTTMs) have been carried out comparing the properties as well as the extraction process employing liquid‐liquid (micro)extraction in glass and polypropylene materials. We delve into the intriguing world of magnetic mixtures and explore how their behavioral changes depend on the surface nature of the material in which they are prepared, stored and used. By understanding the impact of the support material, we can enhance not only the efficiency of magnetic‐based separation processes but also unlock the full potential of these novel mixtures.

## Results and Discussion

### MDESs and MLTTMs Behavior Depending on the Container Surface Material

At this stage, we envisioned to perform microextractions using different MDESs and MLTTMs [i. e., FeCl_3_ ⋅ 6H_2_O : Ethylene glycol (1 : 2), CoCl_2_ ⋅ 6H_2_O : Ethylene glycol (1 : 2), NiCl_2_ ⋅ 6H_2_O : Ethylene glycol (1 : 2) and Choline chloride:Ethylene glycol:FeCl_3_ ⋅ 6H_2_O (1 : 4 : 1)] as a magnetic solvent for the extraction and preconcentration of different organic and inorganic analytes. In order to prepare all the mixtures employed during the experiments, ethylene glycol (EG, Sigma‐Aldrich, anhydrous, 99.8 %, Saint Louis, MO, United States), CoCl_2_ ⋅ 6H_2_O (Alfa Aesar, 98 %, Kandel, Germany), FeCl_3_ ⋅ 6H_2_O (Fluka, 98 %, Buchs, Switzerland), choline chloride (Sigma‐Aldrich, anhydrous, 98 %) and NiCl_2_ ⋅ 6H_2_O (Panreac, 97 %, Barcelona, Spain) were used. Once the mixtures were prepared, the water content present in each of them was determined using Karl‐Fischer titration (KF V20 Compact, Mettler Toledo, Columbus, OH, United States) obtaining water content percentages of 13.4±0.3 % for FeCl_3_ ⋅ 6H_2_O : Ethylene glycol (1 : 2), 25.9±0.6 % for CoCl_2_ ⋅ 6H_2_O : Ethylene glycol (1 : 2), 27.9±0.2 % for NiCl_2_ ⋅ 6H_2_O : Ethylene glycol (1 : 2), and 9.0±0.1 % for Choline chloride:Ethylene glycol:FeCl_3_ ⋅ 6H_2_O (1 : 4 : 1).

**Figure 1:**
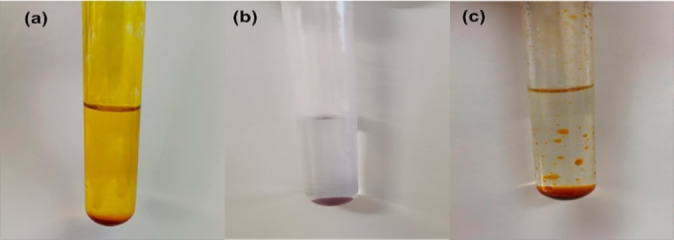
Magnetic mixture [choline chloride:ethylene glycol:FeCl_3_ ⋅ 6H_2_O (1 : 4 : 1)] behavior after dispersion depending on the nature of material used: a) glass tube; b) polypropylene tube; and c) deactivated glass tube.

When trying to carry out the dispersion of the solvent [Choline chloride:Ethylene glycol:FeCl_3_ ⋅ 6H_2_O (1 : 4 : 1)] in 10 mL glass tubes (mainly SiO_2_) (Análisis vínicos, Tomelloso, Spain), using a vortex (Reax Top, Heidolph Instruments, Schwabach, Germany) as a mechanical agitation, it should be noted that magnetic solvent were not dispersed properly since the solvent was homogeneously adhered to the whole wall of the tube, as can be seen in Figure 1a. In addition, it is possible to observe a loss of magnetism of magnetic mixture since the phase separation with an external magnet (Supermagnete, Gottmadingen, Germany) is a time‐consuming process (Videos S1 and S2). This may be explained by the fact that the structure of the magnetic mixture is modified in contact with glass surface. Therefore, we attempted the same dispersive liquid‐liquid microextraction procedure in 10 mL plastic tubes (i. e., polypropylene) (Deltalab, Barcelona, Spain) observing that the behavior of the magnetic mixtures was completely different in this material, maintaining the magnetic properties and the phases separation takes place immediately (Figure 1b). In this case, neither adhesion to the tube walls, change of the structure nor loss of the magnetism was detected (Videos S3 and S4), which facilitates the extraction process with the collection of MDES (or MLTTM) with an external magnetic field. Additionally, a glass deactivation process of the glass tubes was carried out following the methods described in the literature.[^13^, ^14^] In this way, the surface of the tubes was modified with Me_3_SiCl and not as much adhesion effects and loss of magnetism were observed compared to the original glass tubes (Figure 1a), as depicted in Figure 1c.

### Surface Tension and Contact Angle

The surface tension of the above‐mentioned magnetic mixtures was measured as can be seen in Table 1. The surface tension of inorganic salts‐based mixtures is significantly high (comparable to water which is 72.8 mN m^−1^, 20 °C), which might be due to strong hydrogen bond interactions between the components. This fact makes sense if we compare the results obtained with those reported by literature.^[15]^ In that study the surface tension of several mixtures was measured, including FeCl_3_ ⋅ 6H_2_O : Ethylene glycol (2 : 1) and Choline chloride:Ethylene glycol:FeCl_3_ ⋅ 6H_2_O (1 : 2:0.6) showing much lower values than those reported in our study (Table 1). This may be mainly due to the increase in the molar ratio of the ethylene glycol in our cases (the HBD), which is well known for forming hydrogen bong networks via the OH, thus incrementing the surface tension values.^[15]^

**Table 1:** Surface tension and contact angle on glass and polypropylene surfaces of the different magnetic mixtures.

Magnetic mixture	Surface tension (mN m^−1^)	Contact angle (°)	
		Glass	Polypropylene
FeCl_3_ ⋅ 6H_2_O : Ethylene glycol (1 : 2)	71.8±0.1	<90	<90
CoCl._2_ ⋅ 6H_2_O : Ethylene glycol (1 : 2)	77.8±0.5	<90	>90
NiCl_2_ ⋅ 6H_2_O : Ethylene glycol (1 : 2)	77.5±0.1	<90	<90
Choline chloride:Ethylene glycol:FeCl_3_ ⋅ 6H_2_O (1 : 4 : 1)	68.2±0.3	<90	<90
FeCl_3_ ⋅ 6H_2_O : Ethylene glycol (2 : 1)*	57.0		
.Choline chloride:Ethylene glycol: FeCl_3_ ⋅ 6H_2_O (1 : 2 : 0.6)*	50.1		

*From reference [15].

The surface tension of the mixtures was measured at 22 °C on a Phywe equipment (Göttingen, Germany) using a 19.5 mm diameter metal ring and the DuNouy ring method was used. For each mixture, three replicates were measured and averaged. Photos of droplets on the glass and polypropylene surfaces (thickness: 150 μm) were taken at 21 °C on a goniometer (GBX Instruments, Bourg de Pèage, France). Droplets of 3 μL of the different magnetic mixtures were placed using a micropipette in different areas of the glass or polypropylene surface.

**Figure 2:**
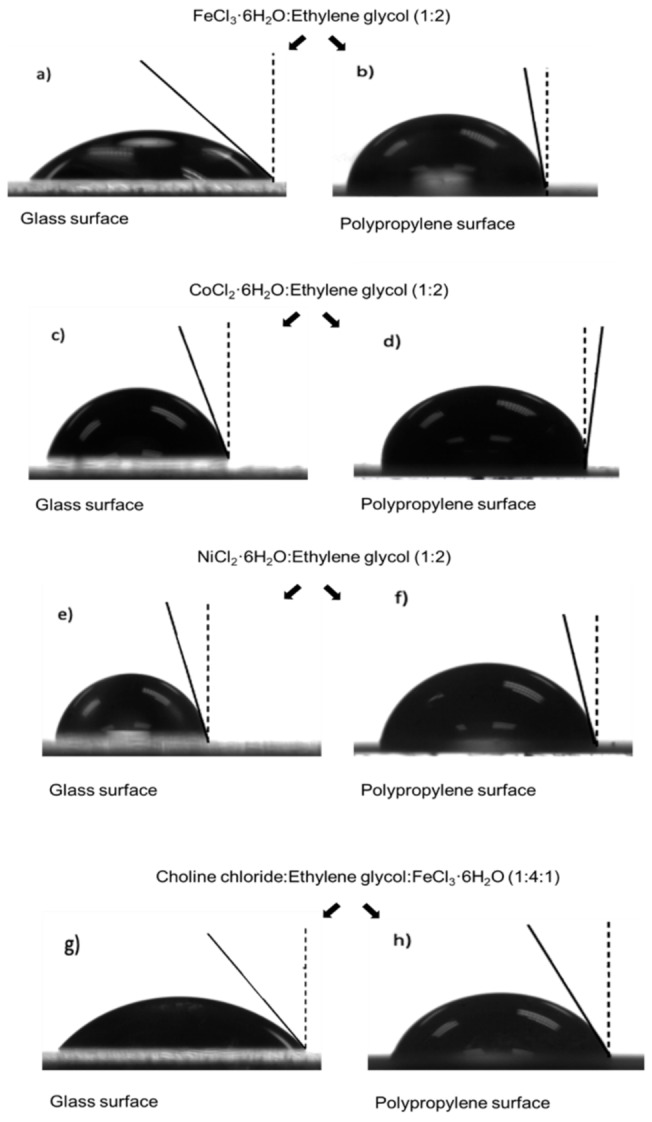
Pictures of the magnetic mixtures, showing the contact angles with polypropylene and glass material. A) FeCl_3_ ⋅ 6H_2_O : Ethylene glycol (1 : 2) in glass surface; b) FeCl_3_ ⋅ .6H_2_O : Ethylene glycol (1 : 2) in polypropylene surface; c) CoCl_2_ ⋅ 6H_2_O : Ethylene glycol (1 : 2) in glass surface; d) CoCl_2_ ⋅ 6H_2_O : Ethylene glycol (1 : 2) in polypropylene surface; e) NiCl_2_ ⋅ 6H_2_O : Ethylene glycol (1 : 2) in glass surface; f) NiCl_2_ ⋅ 6H_2_O : Ethylene glycol (1 : 2) in polypropylene surface; g) Choline chloride:Ethylene glycol:FeCl_3_ ⋅ 6H_2_O (1 : 4 : 1) in glass surface; h) Choline chloride:Ethylene glycol:FeCl_3_ ⋅ 6H_2_O (1 : 4 : 1) in polypropylene surface.

Furthermore, the contact angles of the MDES (or MLTTMs) were measured on different surfaces (i. e., glass and polypropylene) showing the different wettability of these magnetic solvents depending on the surface in which they are placed. As shown in Figure 2, the contact angles of the magnetic mixtures with the material are below 90° when placed in glass materials. In contrast, when the magnetic mixtures are placed on a polypropylene surface, the contact angles are higher in all cases compared with glass surfaces, showing poor wettability between both materials (i. e., MDESs/MLTTMs and polypropylene). Considering these dissimilarities, it is possible to understand the different behaviors of the magnetic mixtures when the dispersion was performed in glass and polypropylene tubes as shown in Figure 1 and Videos S1–S4.

### Thermal Behavior of the Magnetic Mixtures

In order to corroborate the thermal nature of the substances used, several magnetic mixtures, apart from those studied in the previous section, were prepared and their thermal behavior was studied using Differential Scanning Calorimetry (DSC) technique (Table 2 and Figures S1–S12). In general, all the magnetic mixtures present a glass transition temperature (*T_g_*) in the thermograms instead of a well‐defined melting point, therefore they could be properly described as MLTTMs.^[16]^

**Table 2:** Thermal analysis of different magnetic mixtures composed by a metallic halide and ethylene glycol.

Composition	State after 24 h	Thermal behavior	Type of solvent
FeCl_3_ ⋅ 6H_2_O : EG^[a]^ (1 : 2)	Liquid	*T_g_* (−11.7±1.5 °C)	MLTTM
FeCl_3_ ⋅ 6H_2_O : EG (1 : 1)	Liquid	*T_g_* (−23±2 °C)	MLTTM
FeCl_3_ ⋅ 6H_2_O : EG (2 : 1)	Semi solid	*T_g_* (−12±3 °C)	MLTTM
FeCl_3_ ⋅ 6H_2_O : EG (1 : 3)	Liquid	*T_g_* (−20±7 °C)	MLTTM
CoCl_2_ ⋅ 6H_2_O : EG (1 : 2)	Liquid	*T_g_* (−30±6 °C)	MLTTM
CoCl_2_ ⋅ 6H_2_O : EG (1 : 1)	Liquid	*T_g_* (−37±3 °C)	MLTTM
CoCl_2_ ⋅ 6H_2_O : EG (2 : 1)	Semi solid	*T_g_* (−21±5 °C)	MLTTM
.CoCl_2_ ⋅ 6H_2_O : EG (1 : 3)	Liquid	–^[b]^	–
NiCl_2_ ⋅ 6H_2_O : EG (1 : 2)	Liquid	*T_g_* (−32.05 °C)	MLTTM
NiCl_2_ ⋅ 6H_2_O : EG (1 : 1)	Semi Liquid	–	–
NiCl_2_ ⋅ 6H_2_O : EG (2 : 1)	Solid	*T_g_* (−48.22 °C)	MLTTM
NiCl_2_ ⋅ 6H_2_O : EG (1 : 3)	Liquid	–	–

[a] EG: ethylene glycol. [b] (−): no thermal events.

For DSC measurements the MDESs and MLTTMs were prepared at atmospheric pressure mixing the corresponding metallic halide and ethylene glycol at different ratios. Each one was heated up to 60–80 °C for 30 minutes until a clear solution appears. All of them remain liquid (or semi solid) after 24 hours.

All mixtures were placed in a hermetic aluminum standard melting pot. An identical pan and lid were used to carry out the reference in absence of the mixture. All experiments were performed on a Mettler Toledo equipment (Mettler Toledo), model TGA/SDTA851e/LF/1600. In DSC experiments, the mixtures were continuously purged with 50 mL min^−1^ of nitrogen. Around 10 mg of each mixture was crimped in the pan and analyzed under dynamic nitrogen atmosphere by heating (5 °C min^−1^) and cooling (5 °C min^−1^) cycles between −70 and 120 °C. In all the DSC curves the exothermic transitions are shown up while the endothermic ones down.

The vast majority of exothermic events detected in the thermograms during the first cooling/heating cycle can be attributed to either kinetic event or to an increase in the pressure of the system since the pots were slightly deformed. These exothermic events may partially represent the decomposition of the mixtures. The glass transition temperature (*T_g_*) is observed in the second heating cycle. For greater clarity of the results, the thermograms of the FeCl_3_ ⋅ 6H_2_O : Ethylene glycol and CoCl_2_ ⋅ 6H_2_O:Ethylene glycol mixtures present this second heating cycle in triplicate, without considering the first cooling/heating cycle as generally it is attributed to some kinetic events (Figures S1–S8).

**Figure 3:**
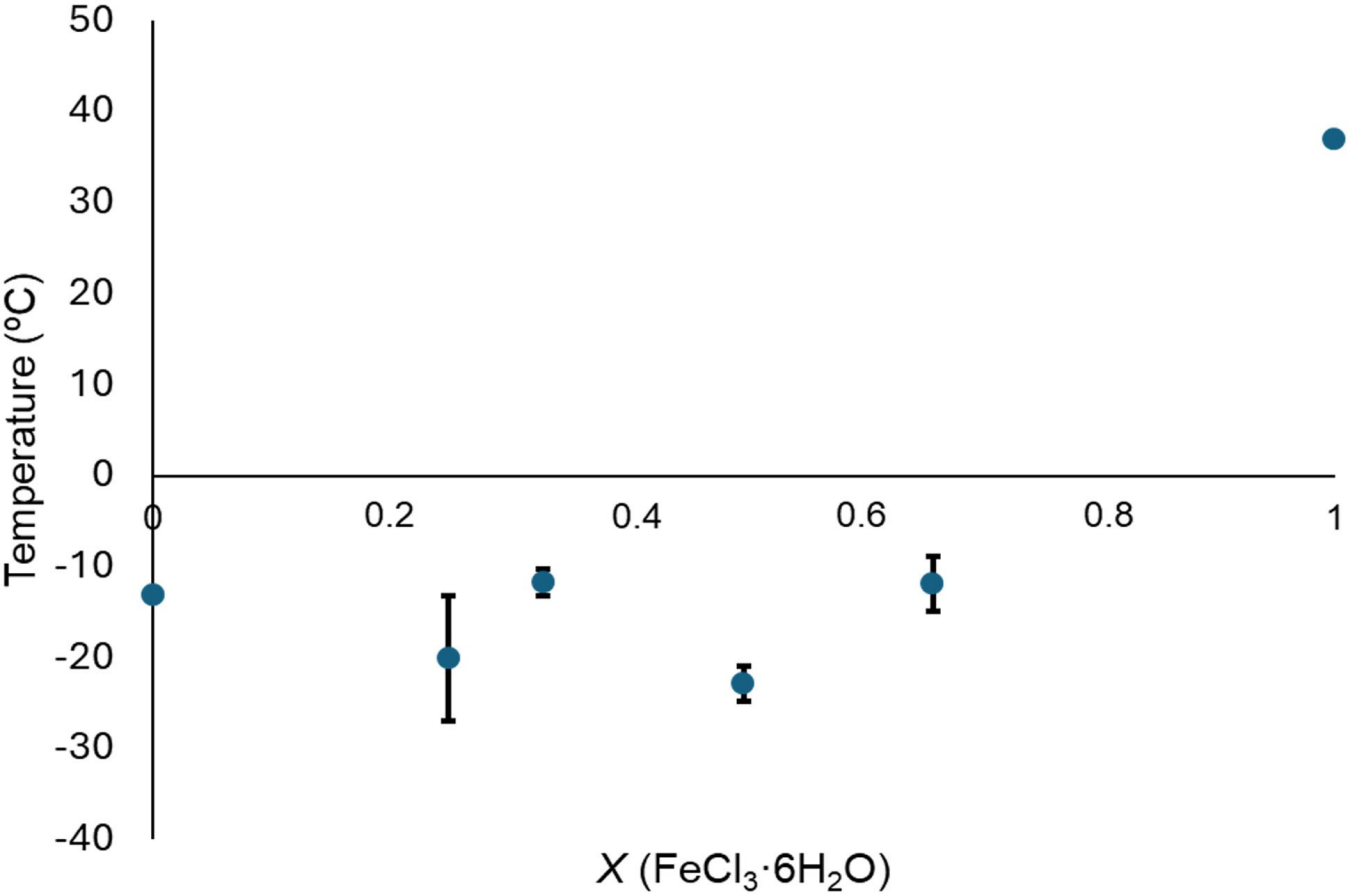
Phase diagram for the magnetic mixtures composed by FeCl_3_ ⋅ 6H_2_O and ethylene glycol.

**Figure 4:**
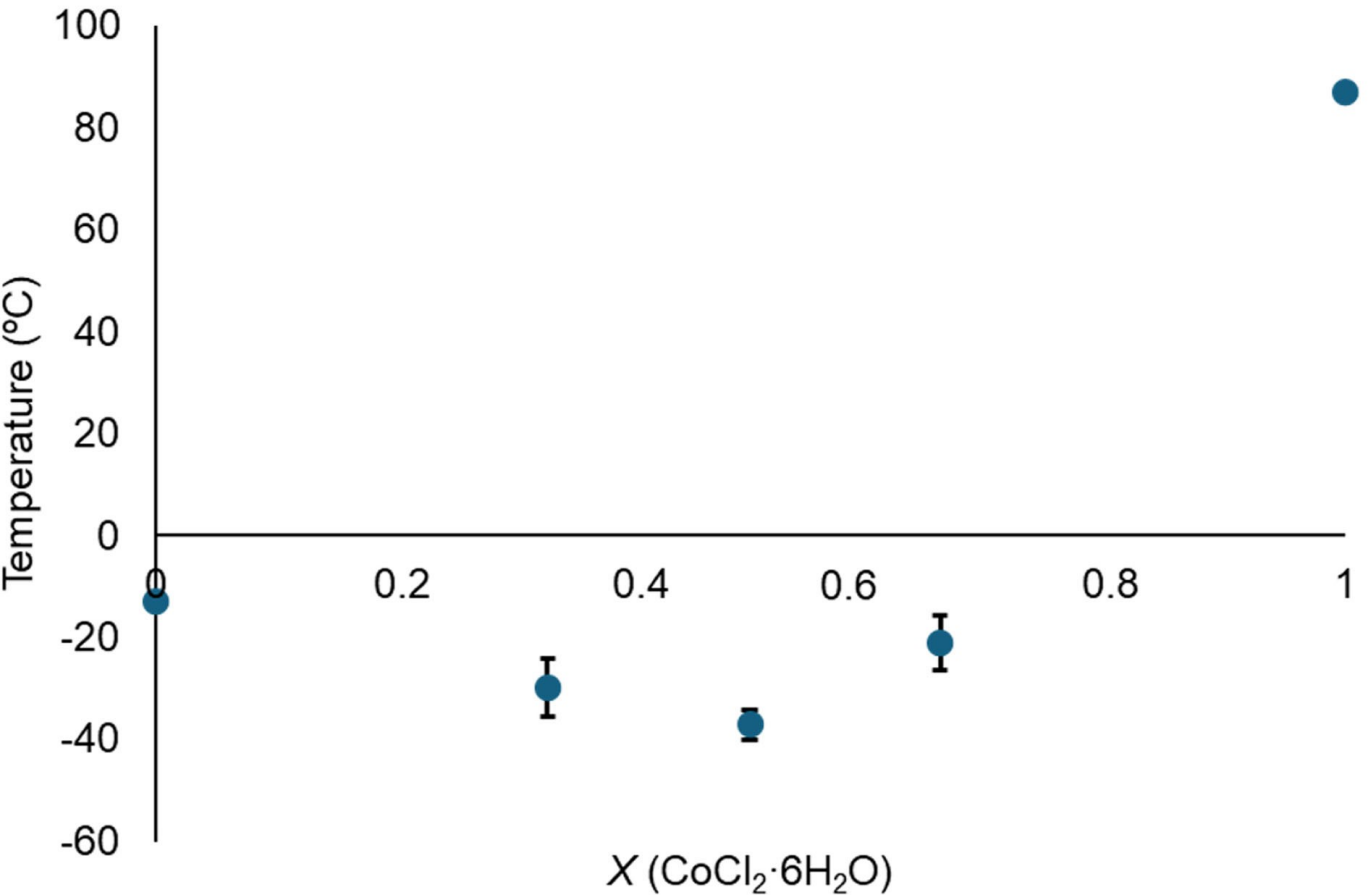
Phase diagram for the magnetic mixtures composed by CoCl_2_ ⋅ 6H_2_O and ethylene glycol.

Additionally, some obtained phases diagrams were plotted for the magnetic mixtures composed by FeCl_3_ ⋅ 6H_2_O and ethylene glycol (Figure 3) and for CoCl_2_ ⋅ 6H_2_O and ethylene glycol (Figure 4). In the former, two minimum temperatures were detected for the magnetic mixtures with the composition FeCl_3_ ⋅ 6H_2_O : Ethylene glycol (1 : 1) and (1 : 3). Nevertheless, no thermal behavior was observed for the magnetic mixtures composed by CoCl_2_ ⋅ 6H_2_O and ethylene glycol (1 : 3) (Figure S8), and the two minimum temperatures were found for molar ratios (1 : 1) and (1 : 2).

### SEM‐EDX Analyses

**Figure 5:**
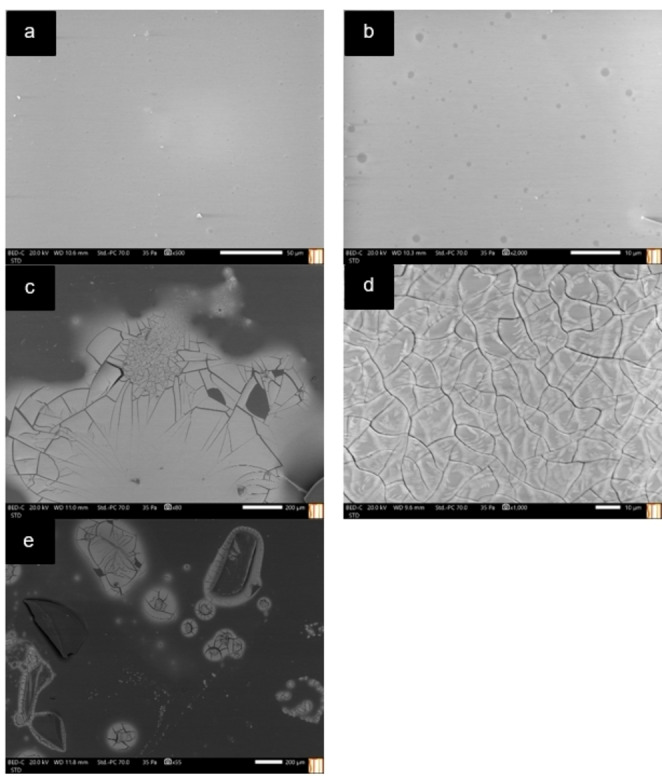
SEM images of the analyses in a) and b) non‐used glass tube, c), d) and e) used glass tube with FeCl_3_ ⋅ 6H_2_O : Ethylene glycol (1 : 2) magnetic mixture.

**Figure 6:**
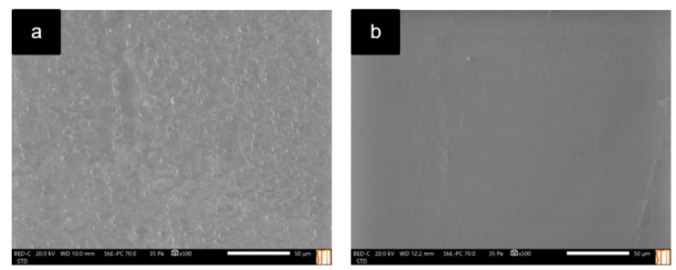
SEM images of the analyses in a) non‐used polypropylene tube, b) used polypropylene tube with FeCl_3_ ⋅ 6H_2_O : Ethylene glycol (1 : 2) magnetic mixture.

The surface of both the glass and polypropylene tubes were studied by scanning electron microscopy coupled with energy dispersive X‐ray spectroscopy (SEM‐EDX) (Figures 5, 6, and S13–S16). In the analysis the mixtures were placed in glass and plastic tubes (i. e., polypropylene) performing a dispersion with vortex agitator. Then, the glass surfaces were carefully broken with a hammer and directly introduced in the equipment to avoid any possible crossed contamination broken with a hammer and directly introduced in the equipment. In the case of the polypropylene tubes, they were cut in pieces using scissors and introduced in the equipment. Finally, the polypropylene and glass tubes with and without performing the dispersion of the mixtures (i. e., MDES and MLTTM) were analyzed by Hitachi brand scanning electron microscope model S3000 N (Hitachi, Tokio, Japan). This microscope has a Bruker model XFlash 3001 X‐ray detector for microanalysis in EDX and mapping (Bruker, Billerica, MA, United States). It is important to mention that the glass tubes were analyzed in triplicate while the plastic surfaces could only be measured once due to the material was melt after one measurement due to the energy applied.

Figure 5a and b show the morphology of two different positions of a surface of a non‐used glass tube being both the expected for an aluminosilicate material. Information on the elemental composition of non‐used glass tubes was verified by the EDX and can be found in Figure S13. In contrast, the surface of the tube that had been in contact with the FeCl_3_ ⋅ 6H_2_O : Ethylene glycol (1 : 2) magnetic mixture showed (after washed with water and acetone) a particular morphology which resembled thin layers of carbon and iron species (Figure 5c–e) in three different positions of the surface. The composition of these layers was verified by the EDX (Figure S14), detecting in all the pieces of the tube a significant percentage of carbon, chlorine and iron which is consistent with the composition of the magnetic mixture used.

Likewise, the same process was followed employing the polypropylene tubes (Figures 6, S15, and S16). The results of SEM experiments observed for the non‐used tube and the tube in which the magnetic mixture was placed were similar (Figure 6). In addition, the composition of the used and non‐used polypropylene tubes was verified by EDX, obtaining similar results in both cases (Figures S15 and S16). These results are evidence of the magnetic mixture interacting with the surface of the glass, changing its intrinsic structure and therefore its magnetic properties. Conversely, no interaction was detected between the polypropylene tube and the magnetic mixture which might explain why the properties of the mixture remain unchanged.

## Conclusions

Different behaviors of magnetic mixtures have been observed depending on the surface nature in which the mixtures are placed. In glass, they tend to adhere to the surface, losing their magnetic properties and losing their capacity to be used as an excellent media for analytical (micro)extraction; this behavior could even be crucial for the use of these mixtures as reaction media. Nevertheless, in plastic surfaces (i. e., polypropylene) a completely different behavior is observed, maintaining their magnetic and structural properties without any adhesion to the surface. These facts have been verified by the surface tension and the contact angle measurements, where a higher contact angle was obtained on the polypropylene surface than on the glass surface for all the magnetic mixtures studied. The thermal behavior of these magnetic mixtures was also studied by DSC proving that they are MLTTMs instead of MDES. Additionally, SEM‐EDX analyses were performed to evidence the adhesion of some metal (i. e., iron) species in the surface of the glass tube in comparison with a polypropylene tube. These results suggest that the MLTTMs might be interacting with the surface of the glass via electrostatic interactions, such as hydrogen bonding or Van der Waals forces, which also might explain the behavior observed in the deactivated glass since less charges are present on the surface. Therefore, there is a possibility that the intrinsic structure of the magnetic mixtures has been disrupted and the individual components are interacting with the surface of the glass, getting attached to it and in consequence changing their properties.

This adhesion to the glass surfaces might explain the significant decrease in the magnetic properties of the mixtures, making difficult the development and validation of new extraction‐based analytical methods. However, the results obtained are a benchmark to new challenges and a plethora of new opportunities to use these magnetic mixtures in analytical applications, by simply replacing the material utilized in the laboratory. In addition, they open the door to novel and thrilling theories about MDESs and MLTTMs structural features and to understand their unique behavior in different surfaces.

The results shown in this work provide some preliminary answers to some observed experimental facts, but most importantly, they suggest many new questions of great importance and significance in many areas of chemistry, from synthetic processes to analytical microextraction. This research began by visually observing the change in the behavior of MDESs/MLTTMs depending on the material of the container when using colored and, in addition, magnetic mixtures. However, many other mixtures do not have one or more of these properties. Therefore, the question we are asking ourselves is: could similar surface nature‐dependent behaviors of (M)DESs and (M)LTTMs have happened in other cases, among the many that have been studied in the different branches of chemistry since the appearance of these new and interesting mixtures? Therefore, the facts presented in this work force us to look back and with new vision at what has been done previously with this type of low volatility mixtures.

## Conflict of Interests

The authors declare no conflict of interest.

1

## Supporting information

As a service to our authors and readers, this journal provides supporting information supplied by the authors. Such materials are peer reviewed and may be re‐organized for online delivery, but are not copy‐edited or typeset. Technical support issues arising from supporting information (other than missing files) should be addressed to the authors.

Supporting Information

Supporting Information

Supporting Information

Supporting Information

Supporting Information

Supporting Information

Supporting Information

Supporting Information

Supporting Information

## Data Availability

The data that support the findings of this study are available in the supplementary material of this article.
