# Supplementary material for: Tunable Properties of Non‐Volatile Magnetic Mixtures on Different Surfaces
Source: Chemphyschem. 2024 Nov 3;25(24):e202400458. doi: 10.1002/cphc.202400458 (PMC11648819; doi:10.1002/cphc.202400458)
Supplement: Supplementary file 1 — Supporting Information [file CPHC-25-e202400458-s004.pdf]

ChemPhysChem

Supporting Information

Tunable Properties of Non-Volatile Magnetic Mixtures on Different Surfaces

Sergio J. Abellán-Martín, Cristina Zapater, Nerea González-Gallardo, Miguel Ángel Aguirre, Lorena Vidal, Diego J. Ramón, and Antonio Canals*

Tunable Properties of Non-Volatile Magnetic Mixtures on Different Surfaces

Sergio J. Abellán-Martín, † [a] Cristina Zapater, † [a] Nerea González-Gallardo,[b] Miguel Ángel Aguirre,[a] Lorena Vidal,[a] Diego J. Ramón,[b] and Antonio Canals*[a]

[a] S.J. Abellán-Martín, C. Zapater, M.Á. Aguirre, L. Vidal and A. Canals
Department of Analytical Chemistry, Nutrition and Food Science and University Institute of Materials
Faculty of Science, University of Alicante
P.O. Box 99, 03080, Alicante, Spain.
†These two authors contributed equally.
*Corresponding author: **a.canals@ua.es**

[b] N. González-Gallardo and D.J. Ramón
Department of Organic Chemistry and University Institute of Organic Synthesis (ISO)
Faculty of Science, University of Alicante
P.O. Box 99, 03080, Alicante, Spain

1. DSC experiments 2
1.1 Some considerations 2
1.2 DSC results 2
2. SEM-EDX Results 9
2.1. Non-used glass tube 9
2.2. Glass tube contaminated with $FeCl_3 \cdot 6H_2O$:EG (1:2) 10
2.3. Non-used polypropylene tube 11
2.4. Polypropylene tube contaminated with $FeCl_3 \cdot 6H_2O$:EG (1:2) 12

1. DSC experiments

1.1 Some considerations

DSC experiments are highly dependent on the kinetics; thus, the freezing point and the melting point are not always equivalent. Several thermal transitions can occur. During cooling, different crystallizations could be noticed. A system could crystallize from an amorphous liquid to a solid structure. It should be notice that when heating crystallization can also happen, since solid-solid transitions can be detected (from an amorphous solid to a more organized one). These transitions are also influenced by the possibility of generating nucleation centers (or stable phases or metaphases). Also, they are highly influenced by the cooling rate since some time is required to organize the crystalline structure. Thus, for instance, if a liquid is cooled quickly the system could have not time enough to organize it leading to a “more amorphous solid”. This could be detected in DSC as the temperature needed would be lower than that of having a highly organized crystalline solid. This also has a significant impact on the heating, as it could determine the possibility of having a melting point or a glass transition temperature.

1.2 DSC results

Figures S1-S12 show DSC curves for all magnetic mixtures studied in this research work.

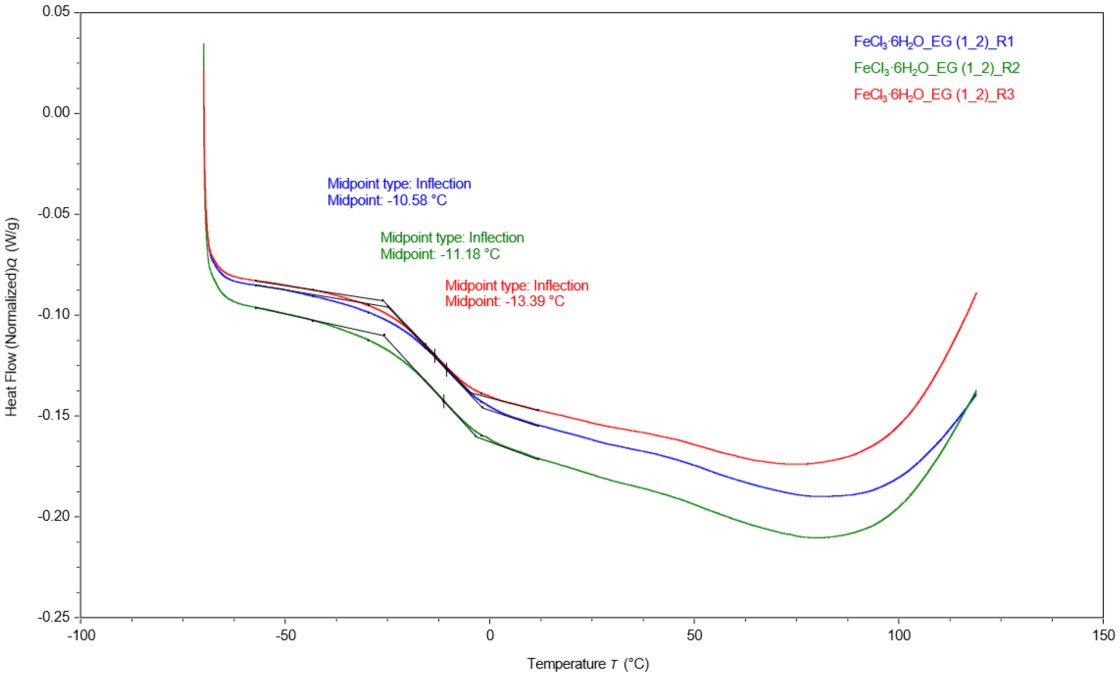

Figure S1. DSC curve for three replicates of the $FeCl_3·6H_2O$:Ethylene glycol (1:2).

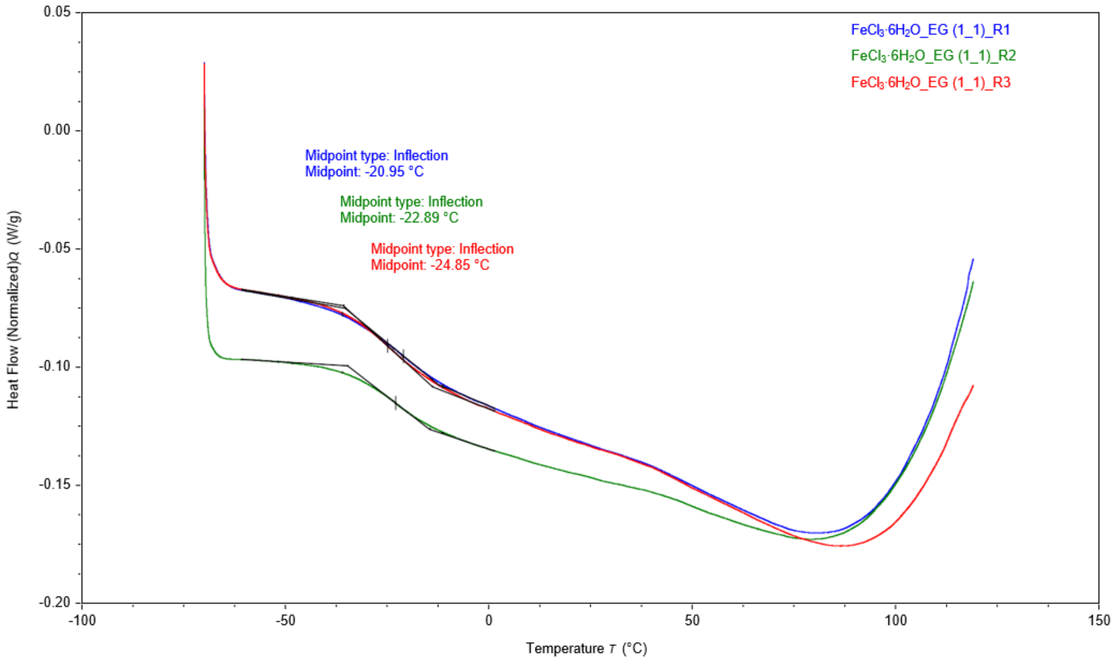

Figure S2. DSC curve for three replicates of the $FeCl_3·6H_2O$:Ethylene glycol (1:1).

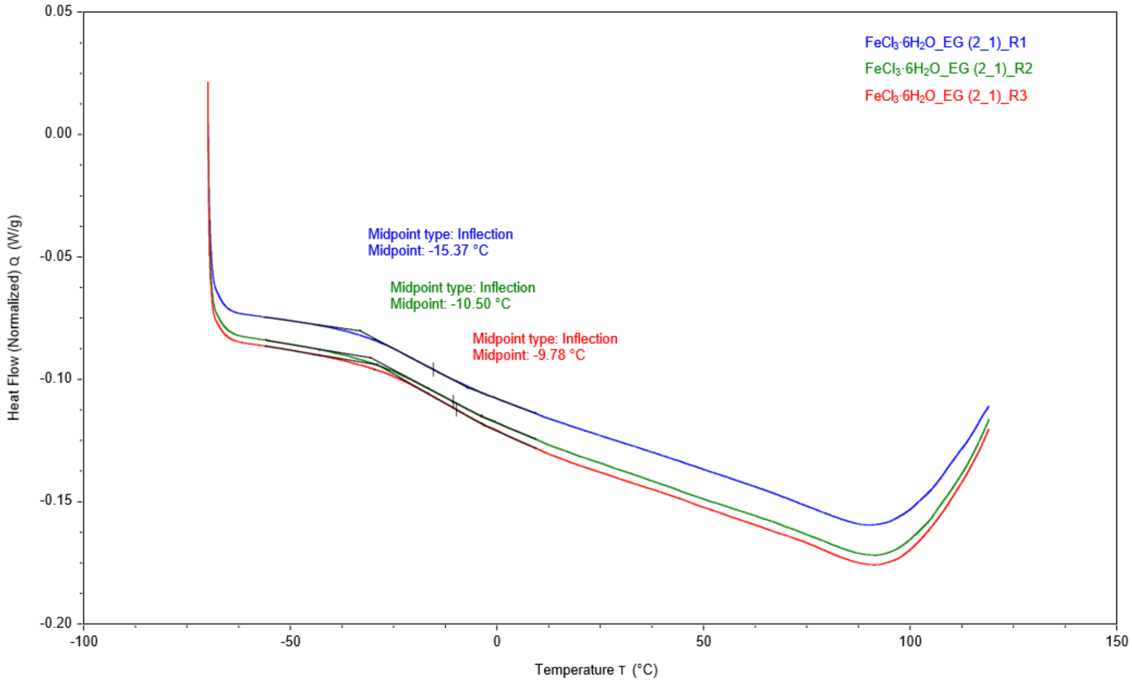

Figure S3. DSC curve for three replicates of the $FeCl_3·6H_2O$:Ethylene glycol (2:1).

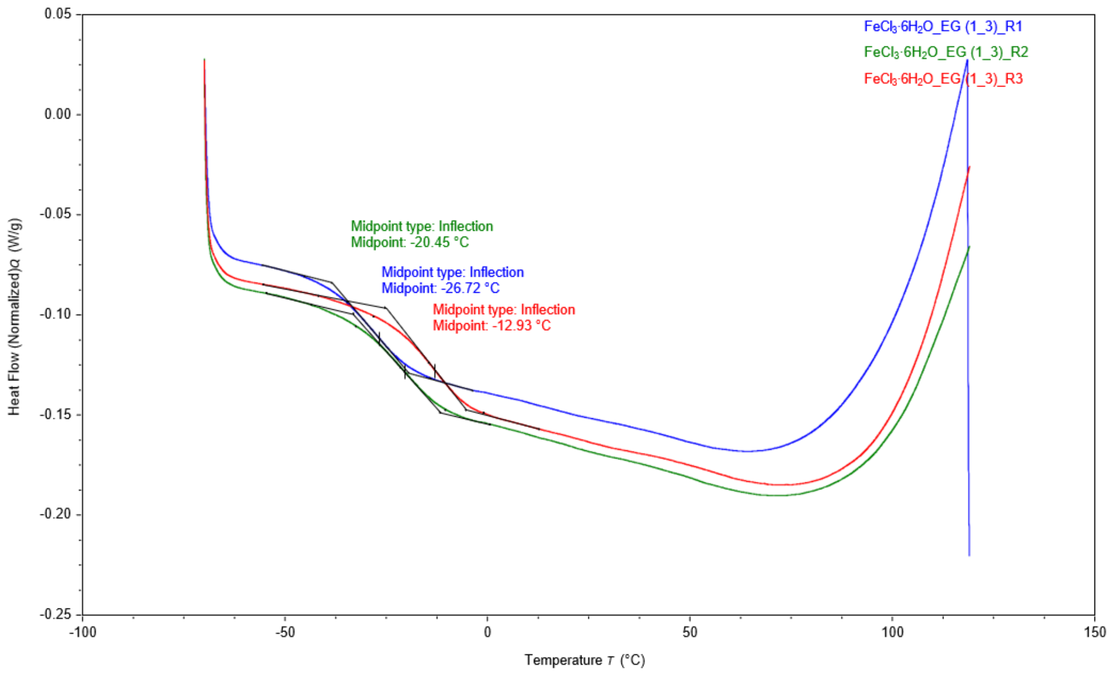

Figure S4. DSC curve for three replicates of the $FeCl_3·6H_2O$:Ethylene glycol (1:3).

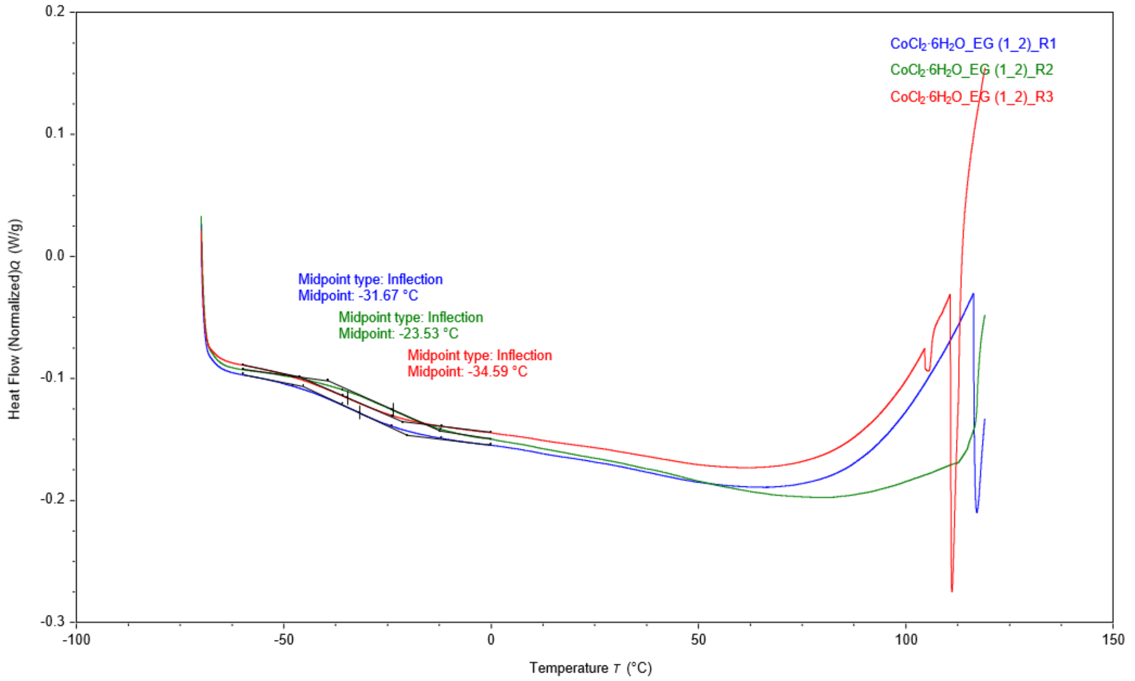

Figure S5. DSC curve for three replicates of the $CoCl_2·6H_2O$:Ethylene glycol (1:2).

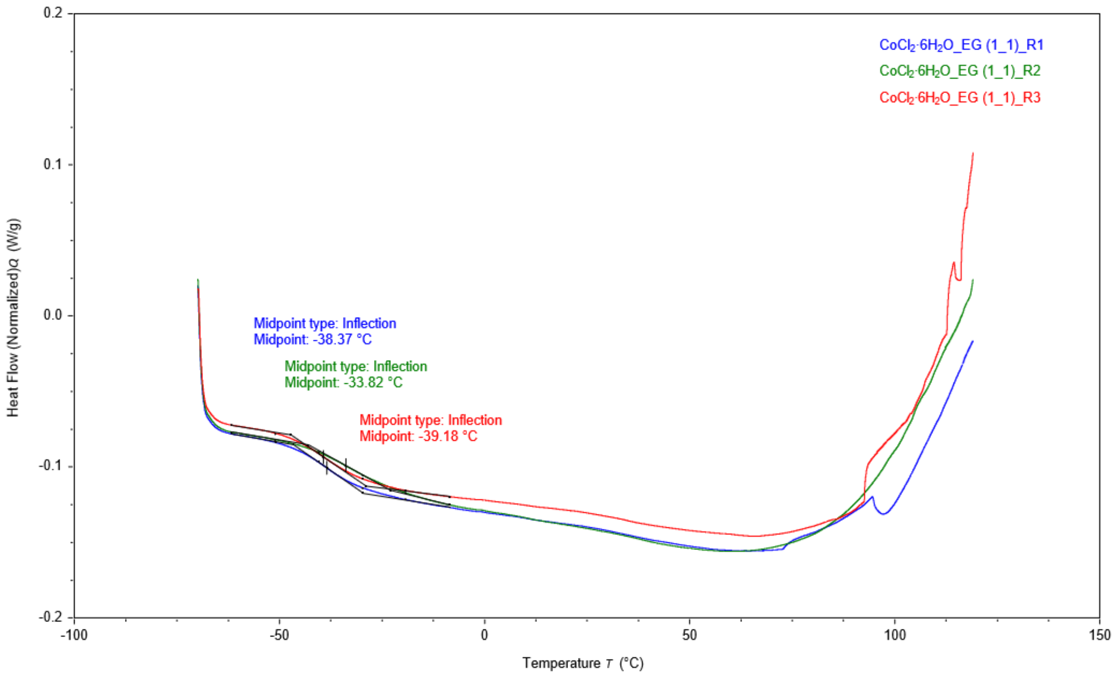

Figure S6. DSC curve for three replicates of the $CoCl_2{\cdot}6H_2O$:Ethylene glycol (1:1).

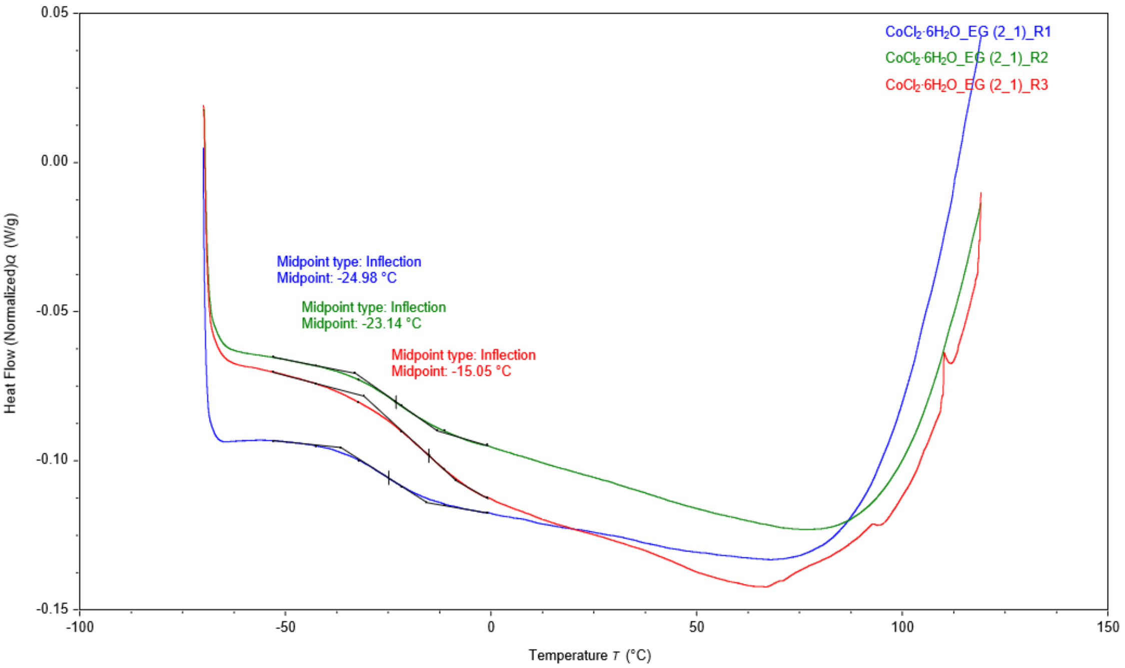

Figure S7. DSC curve for three replicates of the $CoCl_2{\cdot}6H_2O$:Ethylene glycol (2:1).

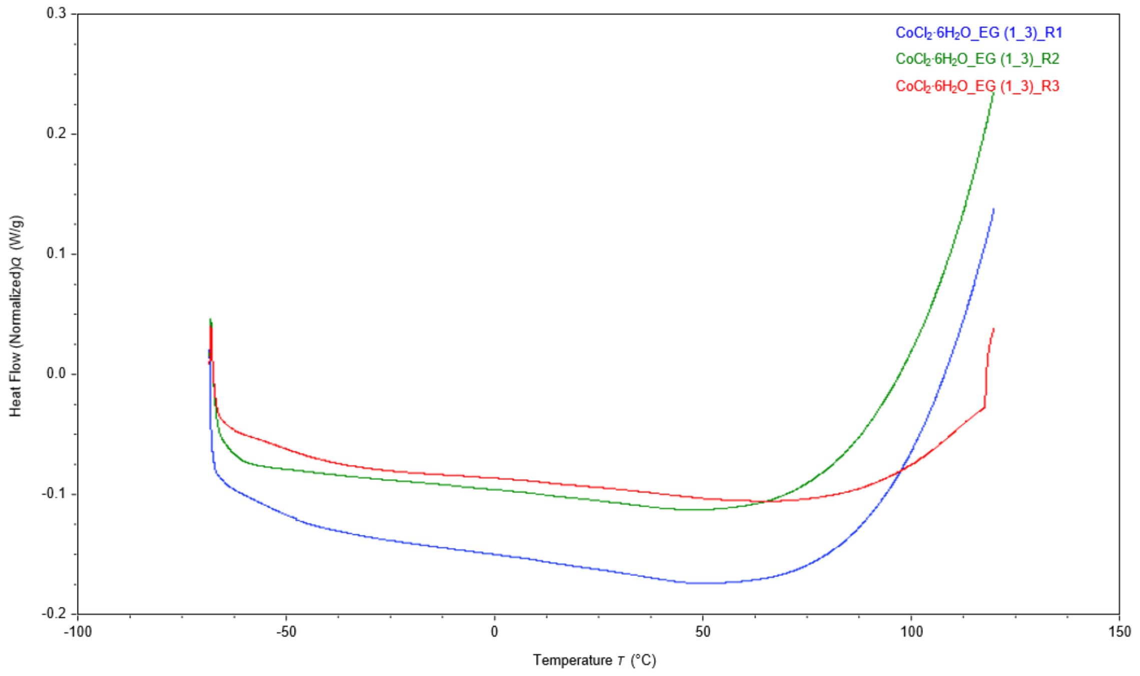

Figure S8. DSC curve for three replicates of the $CoCl_2{\cdot}6H_2O$:Ethylene glycol (1:3).

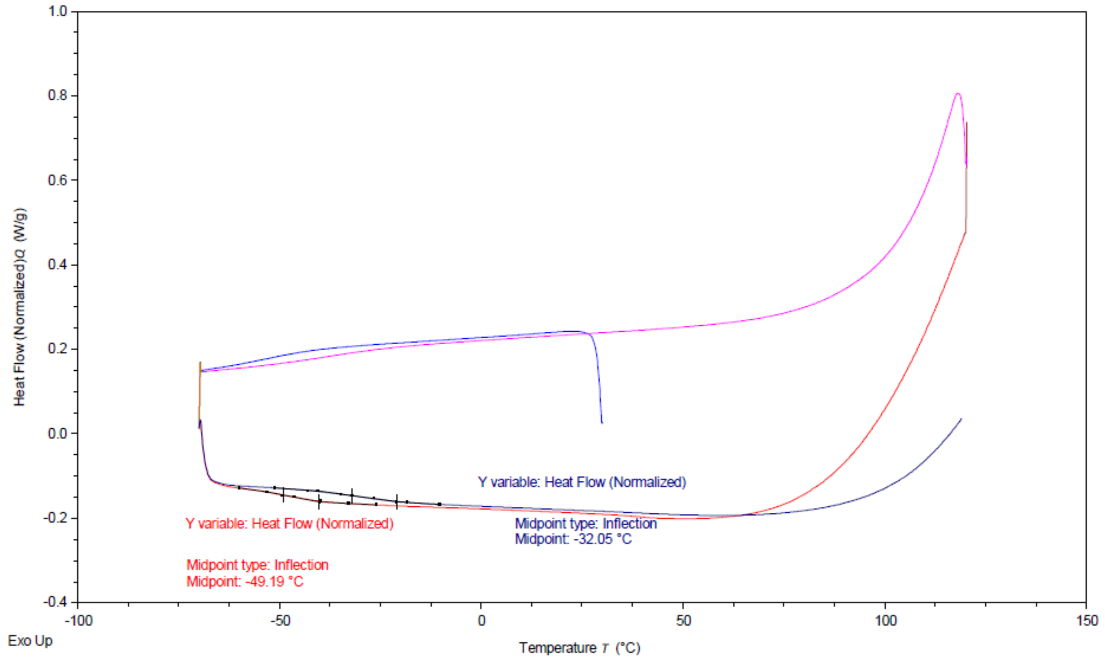

Figure S9. DSC curve of the $NiCl_2{\cdot}6H_2O$:Ethylene glycol (1:2).

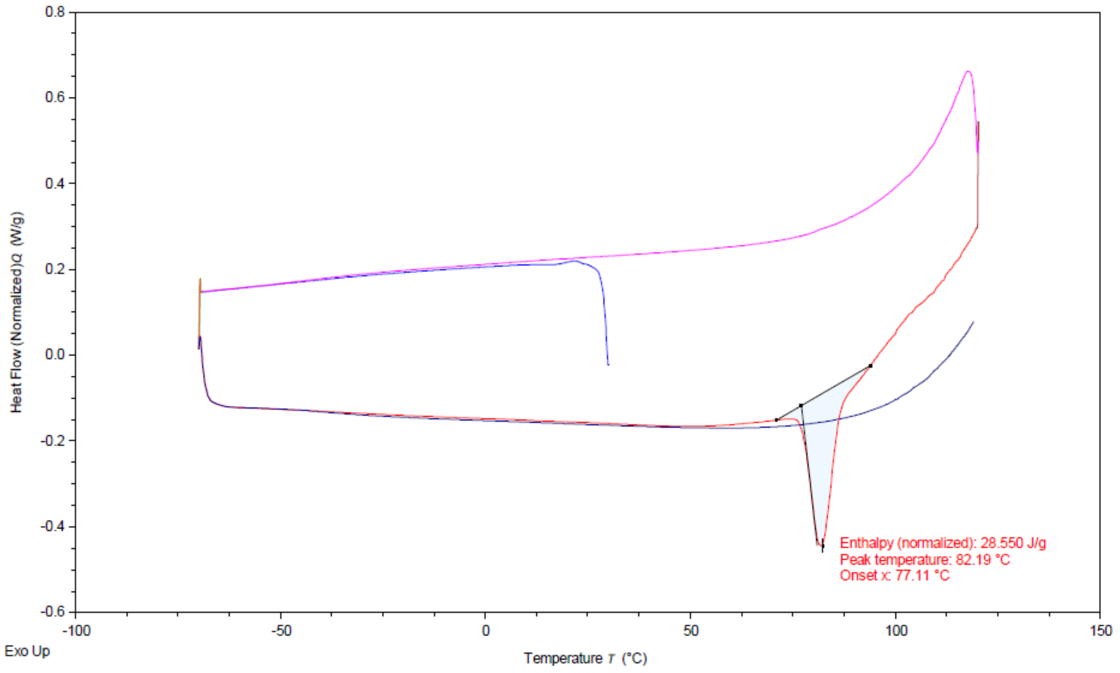

Figure S10. DSC curve of the $NiCl_2 \cdot 6H_2O$:Ethylene glycol (1:1).

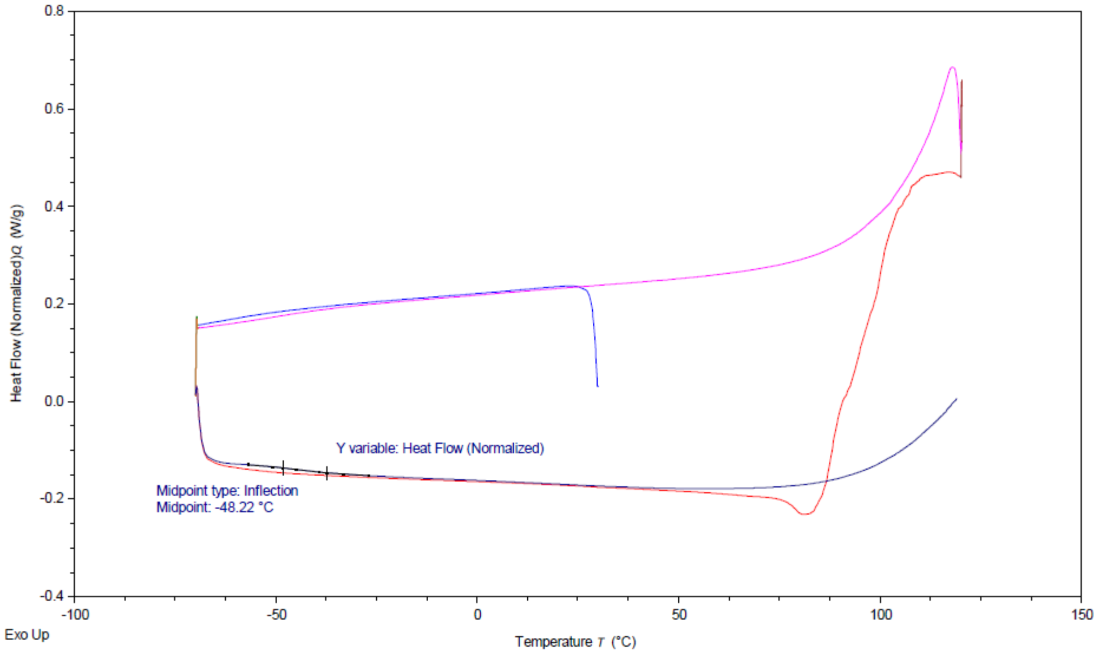

Figure S11. DSC curve of the $NiCl_2 \cdot 6H_2O$:Ethylene glycol (2:1).

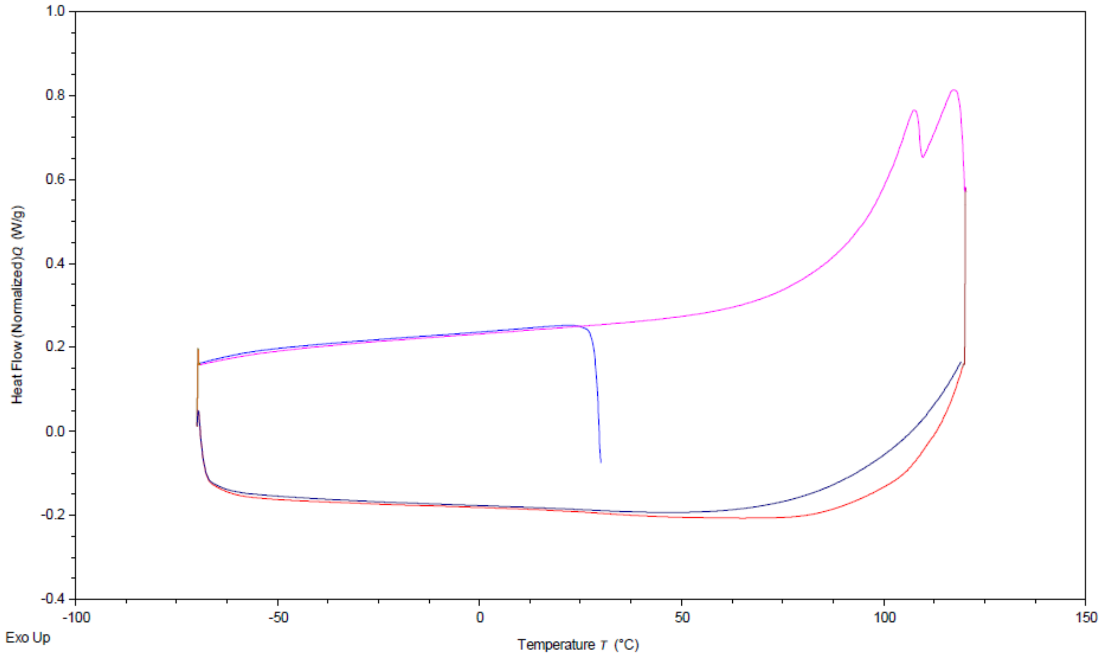

Figure S12. DSC curve of the $NiCl_2 \cdot 6H_2O$:Ethylene glycol (1:3).

2. SEM-EDX Results

2.1. Non-used glass tube

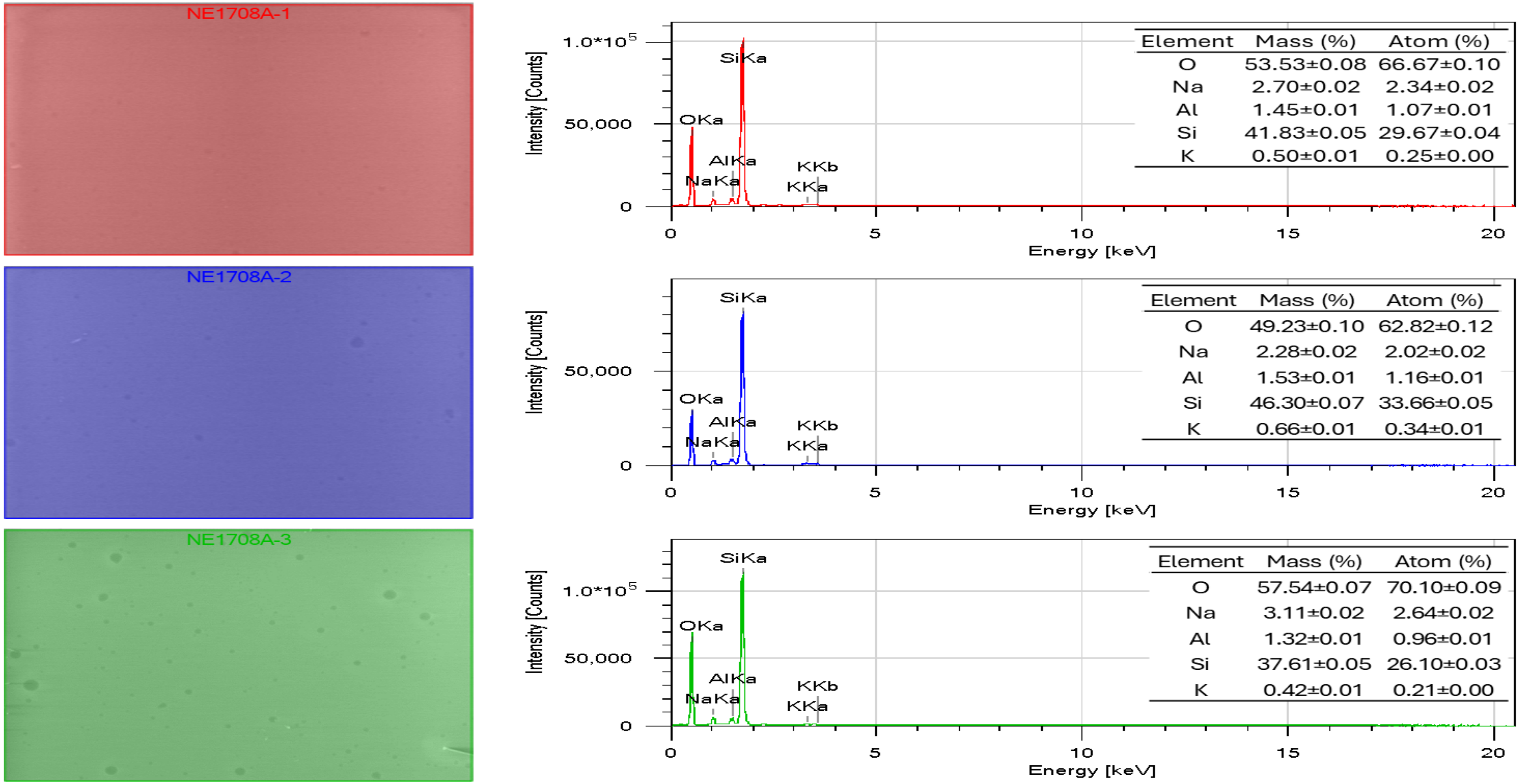

Figure S13. SEM and EDX characterizations of three different positions (i.e., red, blue, and green colors) in non-used glass surface.

2.2. Glass tube contaminated with $FeCl_3 \cdot 6H_2O$:EG (1:2)

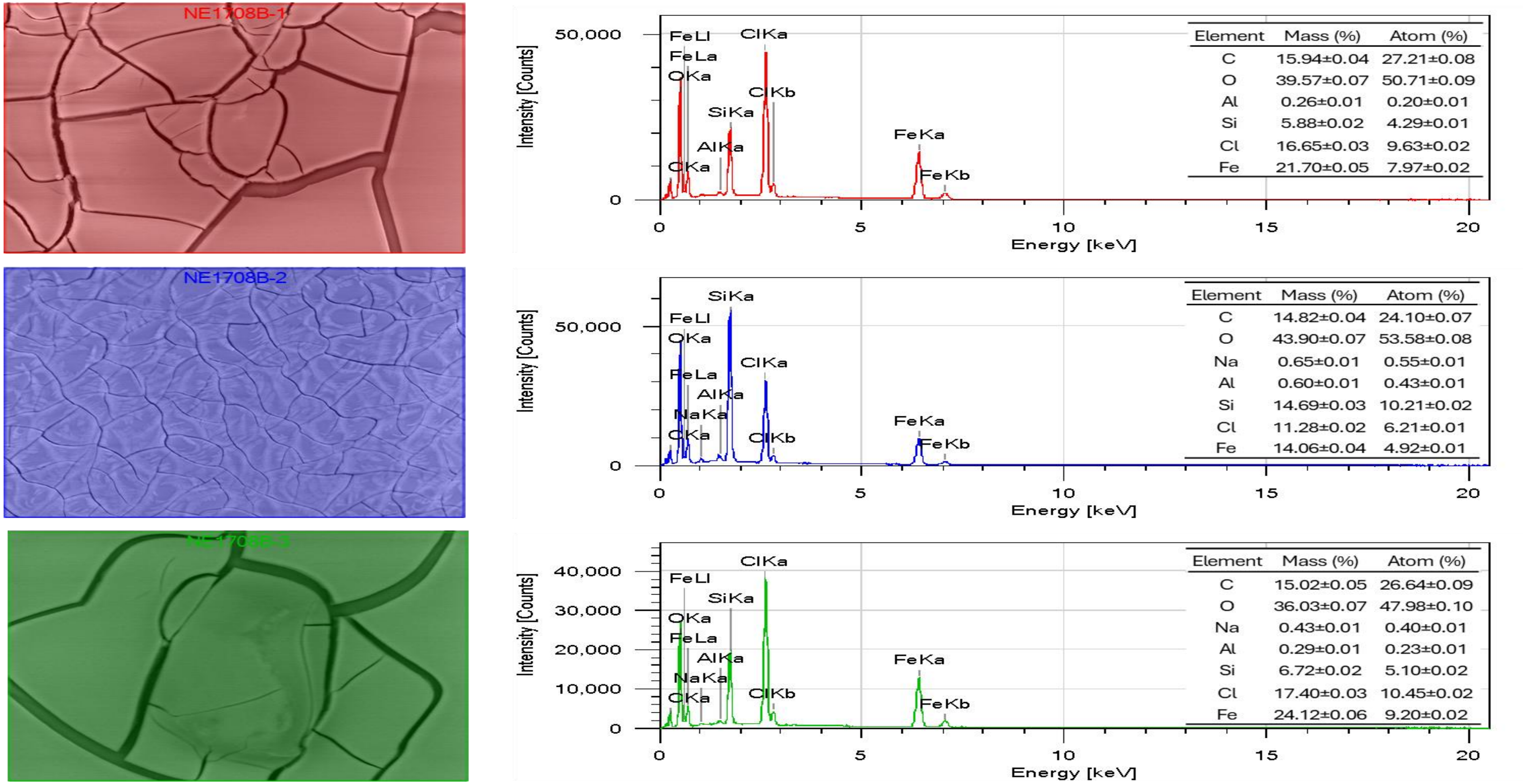

Figure S14. SEM and EDX characterizations of three different positions (i.e., red, blue, and green colors) in used glass surface by $FeCl_3 \cdot 6H_2O$:EG (1:2) after being washed.

2.3. Non-used polypropylene tube

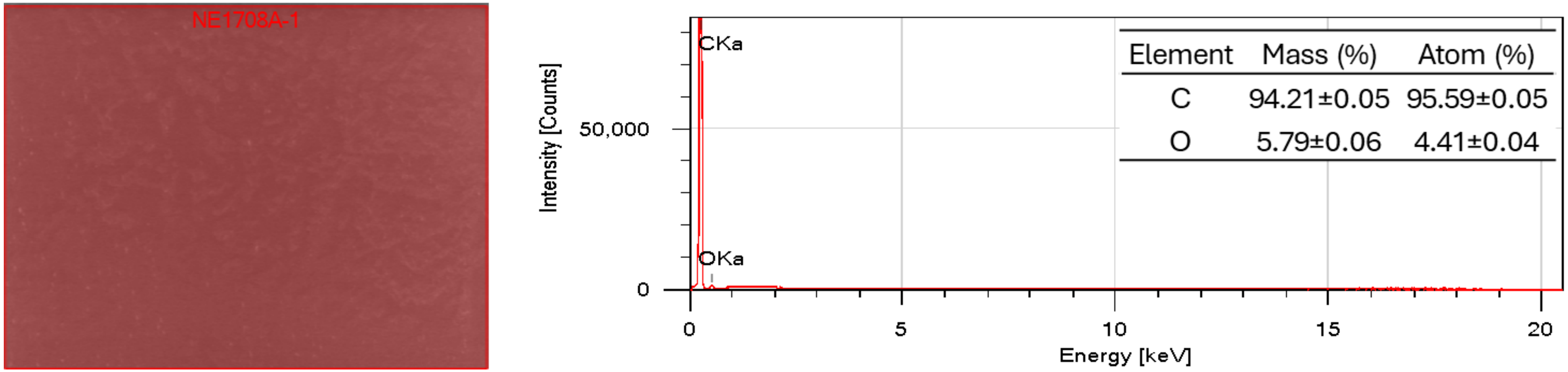

Figure S15. SEM and EDX characterizations at one position in non-used polypropylene surface.

2.4. Polypropylene tube contaminated with $FeCl_3 \cdot 6H_2O$:EG (1:2)

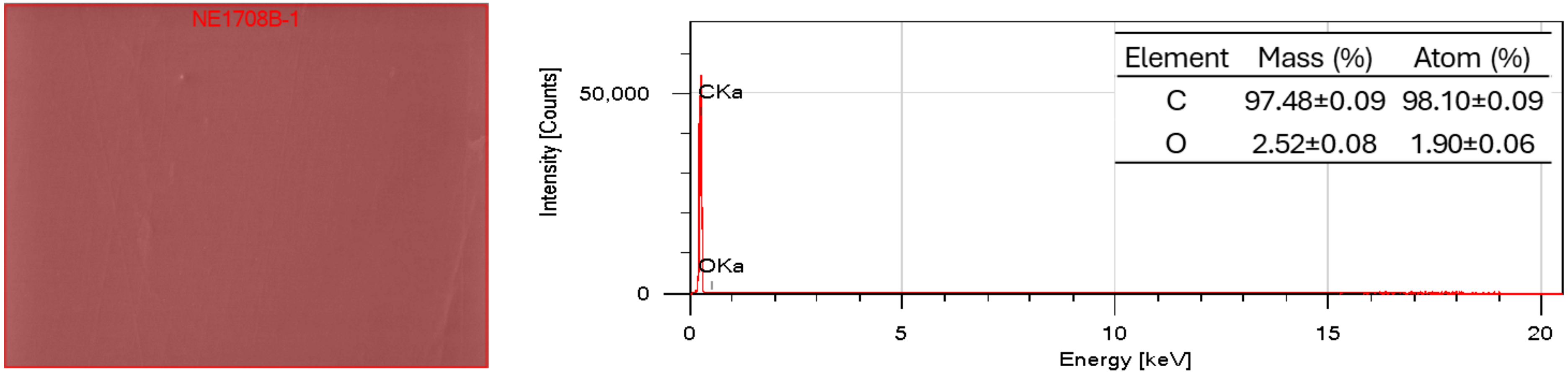

Figure S16. SEM and EDX characterizations at one position in used polypropylene surface by $FeCl_3 \cdot 6H_2O$:EG (1:2) after being washed.
